# Realistic Simulation of Tropical Atmospheric Gravity Waves Using Radar‐Observed Precipitation Rate and Echo Top Height

**DOI:** 10.1029/2019MS001949

**Published:** 2020-07-29

**Authors:** Martina Bramberger, M. Joan Alexander, Alison W. Grimsdell

**Affiliations:** ^1^ NorthWest Research Associates Boulder CO USA

## Abstract

Gravity waves (GWs) generated by tropical convection are important for the simulation of large‐scale atmospheric circulations, for example, the quasi‐biennial oscillation (QBO), and small‐scale phenomena like clear‐air turbulence. However, the simulation of these waves still poses a challenge due to the inaccurate representation of convection, and the high computational costs of global, cloud‐resolving models. Methods combining models with observations are needed to gain the necessary knowledge on GW generation, propagation, and dissipation so that we may encode this knowledge into fast parameterized physics for global weather and climate simulation or turbulence forecasting. We present a new method suitable for rapid simulation of realistic convective GWs. Here, we associate the profile of latent heating with two parameters: precipitation rate and cloud top height. Full‐physics cloud‐resolving WRF simulations are used to develop a lookup table for converting instantaneous radar precipitation rates and echo top measurements into a high‐resolution, time‐dependent latent heating field. The heating field from these simulations is then used to force an idealized dry version of the WRF model. We validate the method by comparing simulated precipitation rates and cloud tops with scanning radar observations and by comparing the GW field in the idealized simulations to satellite measurements. Our results suggest that including variable cloud top height in the derivation of the latent heating profiles leads to better representation of the GWs compared to using only the precipitation rate. The improvement is especially noticeable with respect to wave amplitudes. This improved representation also affects the forcing of GWs on large‐scale circulation.

## Key Points

- Use of an idealized numerical model forced with radar‐derived latent heat profiles to simulate convective gravity waves in the tropics
- Relating latent heat to rain rate and echo top height leads to improved representation of tropical GWs compared to satellite observations
- Approach could improve prediction of tropical gravity waves for weather forecasting and climate applications

## 1 Introduction

Gravity waves (GWs) emanating from convection in the tropics influence atmospheric circulation on different scales. On the large‐scales these are known to drive atmospheric flow patterns such as the quasi‐biennial oscillation (QBO) of the zonal winds of the tropical lower stratosphere (e.g., Alexander & Holton, 1997; Kawatani et al., 2010; Lindzen & Holton, 1968; Ray et al., 1998; Sassi & Garcia, 1994) while on smaller scales the breaking of these waves can cause clear‐air turbulence, which is an acknowledged hazard in aviation (Lane et al., 2012; Sharman & Trier, 2019). For climate prediction and weather forecasting applications, information on GWs from tropical convection is an urgent need (e.g., Beres et al., 2005; Sharman & Trier, 2019). To address these research needs, we present a new method suitable for rapid simulation of realistic GWs emanating from tropical convection, including both developing and more mature storm conditions.

Previous work described model methods for realistic simulations of GWs using scanning precipitation radar data (Stephan & Alexander, 2015; hereafter SA15), and the results have been used to study diverse phenomena ranging from stratospheric GW drag effects in climate models to surface pressure waves and wave influences on convective initiation (Stephan, Alexander, Hedlin, et al., 2016; Stephan, Alexander, & Richter, 2016). The existing method was developed specifically for midlatitude mature continental storm systems during summer conditions.

Applications of the SA15 method have shown that the essential source of GWs within precipitating clouds is the localized and time‐dependent latent heat that is released, and the nonlinear interactions of that heating with the environment. Linear theoretical studies have shown that the depth and time dependence of the heating strongly influence GW properties in the far field (Beres et al., 2004, 2005; Bergman & Salby, 1994; Manzini & Hamilton, 1993). However, the localized, instantaneous latent heating inside convection is highly nonlinear in the sense that the thermodynamic and momentum equations describing the wave generation are coupled through significant momentum and heat flux terms (Song et al., 2003).

Previous modeling studies had found success in matching the important scales observed in the GW field above convection, but had failed in describing the wave amplitudes (e.g., Alexander et al., 2006; Grimsdell et al., 2010). In this context, nonlinear effects within the cloud are known to be quite important for determining wave amplitudes (e.g., Alexander et al., 2006; Chun et al., 2008). The depth of the heating is important in two ways: (1) it projects onto the vertical wave number spectrum of the waves emitted from convection (Alexander et al., 1995; Salby & Garcia, 1987); (2) if the top of the heating extends to a layer of significant shear, the interaction in the shear layer produces a separate class of waves, commonly referred to as the obstacle effect (Alexander et al., 2006; Beres et al., 2004; Chun & Baik, 1998; Pfister et al., 1993).

The SA15 method trained an algorithm using output from nonlinear, full‐physics cloud‐resolving model simulations to characterize the profile of latent heating using a single parameter, the near‐surface precipitation rate. Their method found success in realistic representation of the characteristics of observed wave fields surrounding convective rain in mature midlatitude summertime storm conditions over the continental United States. The method not only gave realistic representation of observed spatial extents and horizontal and vertical wave scales but, uniquely, also gave realistic representation of wave amplitudes. The better agreement in amplitudes was due to stronger estimates of convective latent heating: In accord with an earlier study by Shige et al. (2004), SA15 showed that the latent heating in convective rain cells can be more than a factor of 3 larger than the convective precipitation rates suggest, primarily because much of the condensate formed in convective cells is transported horizontally, where it falls at much weaker rates over large areas. Both Shige et al. (2004) and SA15 also show that latent heating rates increase with rising precipitation rates.

Generally, latent heating is associated with the phase change of water, and consequently, the vertical extent of clouds can be directly connected to the depth and intensity of latent heating in convection. Therefore, in this work, we extend the approach of SA15 and associate the profile of latent heating with two parameters: precipitation rate and echo top height. We train our algorithm on cloud‐resolving model simulations of tropical precipitation in the Darwin, Australia area, where we also have scanning radar observations of precipitation rates and echo top heights available for validation.

We demonstrate the effect of including the additional echo top height variable on the GWs generated by tropical convection. We also show dramatic improvements in the representation of amplitudes of GWs compared to those seen in a satellite overpass. Finally, we compare the effects of the simulated waves on the circulation in the lower stratosphere with and without considering the echo top height variable.

In the following we first provide an overview of the data set taken into account in section 2 followed by a validation of the cloud‐resolving WRF simulations in section 3. In section 4 we present the statistical mean properties of the latent heating profiles, and section 5 shows the GW characteristics based on the different lookup tables. Discussions in section 6 together with conclusions in section 7 close the paper.

## 2 Models and Data

The following study examines convection and GWs from 11 to 13 January 2003 with a focus on 12 January in the area of Darwin, Australia (12.48°S, 130.98°E). Grimsdell et al. (2010) also studied a particularly strong rain event in this period. Here, our modeling approach follows SA15 by employing two different Weather Research Forecasting (WRF) configurations (Skamarock et al., 2008). We first use a conventional cloud‐resolving, full‐physics WRF configuration that is used to define the statistical mean properties of latent heating profiles. The goal of this step is the construction of two different reference tables for the vertical profile of latent heat: One as a function of precipitation rate only, following the SA15 approach, and the other as a function of both precipitation rate and echo top height.

The second WRF configuration is a dry, idealized version of the WRF model. The waves in this model are forced by prescribed latent heating that is based on precipitation rates and echo top heights derived from scanning precipitation radar measurements. The background wind and stability conditions in these idealized simulations are defined by wind and temperature fields from reanalysis fields. The idealized model is extended to high enough altitude that we can sample the wave field with satellite weighting functions for comparisons of observed and simulated waves.

### 2.1 Full‐Physics WRF Simulations

**Figure 1:**
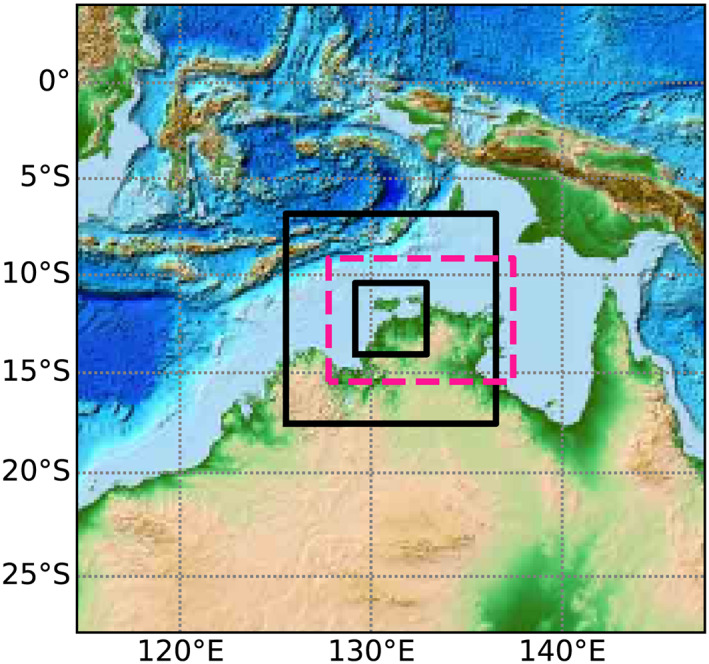
Maps of the different WRF domains. The entire region is contained within the outer domain which has a horizontal grid spacing of 30 km (d01). The inner black boxes show the area covered by the inner two domainswith horizontal grid spacing of 6 km (d02) and 2 km (d03), respectively. The pink dashed line shows the domain of the idealized WRF simulations with a 2‐km horizontal grid spacing.

The cloud‐resolving, full‐physics WRF simulations in this study consist of three nested domains (see Figure  1) connected with a one‐way nesting procedure and were done with WRF version 3.9.1.1. The output frequency of variables is 10 min. The outer domain (d01) spans 3,600 km × 3,600 km, the middle domain (d02) 1,200 km × 1,200 km, and the inner domain (d03) 408 km × 408 km with a horizontal grid spacing of 30, 6, and 2 km, respectively. The horizontal resolution of the inner domain is designed to match the resolution of both the idealized model runs and the resolution of the scanning radar data for the same area. The full‐physics model has a vertical grid consisting of 50 terrain‐following levels. The vertical spacing increases from the ground up to about 7 km, above which the spacing remains fairly constant at about 700 m. A 5 km deep Rayleigh damping layer is used to prevent unphysical reflection of GWs at the upper boundary. The outer domain is initialized at 12:00 UTC on 11 January 2003 with European Centre for Medium‐Range Weather Forecasting (ECMWF) Interim Re‐Analysis (ERA‐Interim) data which also provides boundary conditions throughout the run. The runs end at 12:00 UTC on 13 January 2019. ERA‐Interim data are available at 6 hr interval on 60 vertical levels with a nominal horizontal grid spacing of 0.7° (Dee et al., 2011).

For our study we largely use the “Tropical” WRF physics suite (e.g., Qiao et al., 2019), but with a different surface layer scheme (based on the recommendation of the WRF development team). Our parameterization set includes the WRF Single‐Moment 6‐class (WSM6) microphysics scheme (Hong & Lim, 2006), the Yonsei University planetary boundary layer scheme (Hong et al., 2006), the RRTM (Rapid Radiative Transfer Model) for longwave and shortwave radiation (Iacono et al., 2008; Pincus et al., 2003), and the MM5 (revised fifth‐generation Pennsylvania State University‐National Center for Atmospheric Research Mesoscale Model) surface layer scheme (Jiménez et al., 2012). For the simulation of cumulus clouds the New Tiedke cumulus parameterization scheme (Zhang & Wang, 2017) is used in the outer domain D01. Note no cumulus scheme is used for the inner 6‐km (D02) and 2‐km (D03) domains.

As our study is based on one specific event we use an ensemble of three full‐physics WRF runs to achieve a more statistically robust distribution of the precipitation rates and echo top heights. All three model setups use the same parameterization schemes and initialization data. To achieve spread in the distributions while keeping the initialization as realistic as possible, we vary the height of the model top which slightly changes the vertical resolution. In this study the model tops are 11 hPa (29.8 km), 10 hPa (30 km), and 8.5 hPa (32 km).

### 2.2 Idealized WRF Simulations

**Figure 2:**
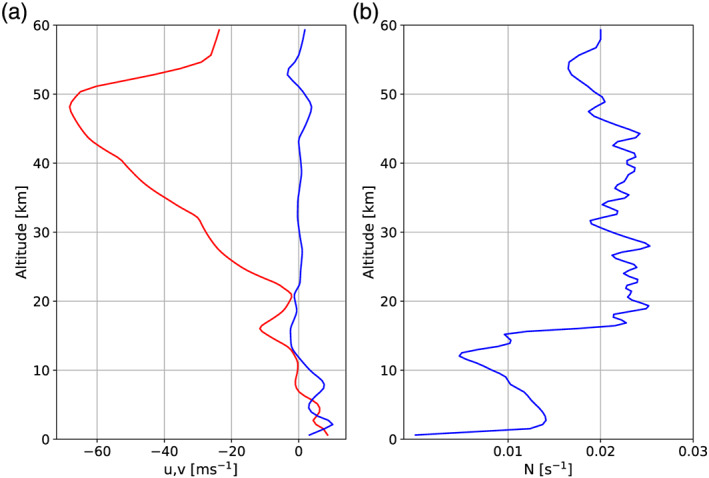
Vertical profiles of the zonal wind speed (*u*, red in panel a), meridional wind speed (*v*, blue in panel a), and static stability (*N*, panel b) used to initialize the idealized WRF simulations.

The idealized WRF model domain covers 700 km in the north‐south dimension (9.15–15.45°S) and 1,050 km in the east‐west dimension (137.44–127.78°E) (see Figure 1). The model domain is larger in the east‐west direction than in the north‐south direction to allow for sufficient space for the mainly eastward stratospheric GWs to propagate (Figure 2a). The grid spacing is 2 km in both horizontal directions and we specify 110 vertical levels up to the model top at 60 km. A 10 km deep Rayleigh damping layer is used in these simulations to prevent unphysical reflection of GWs from the model top. As for the full‐physics simulations, the output frequency of variables is 10 min.

In our study the idealized WRF model is initialized with a temporally and spatially averaged dry sounding of wind and potential temperature from the Modern‐Era Retrospective analysis for Research and Applications 2 (MERRA 2) on 12 January 2003 (see Figure 2). The temporal average is taken over one day and the spatial average is calculated over a latitude range from 14.5°S to 10°S and over a longitude range from 128.75°E to 133.125°E. These locations represent a compromise between the location of the C‐Pol radar and the location of the strongest wave signals in the AIRS measurements we will use for validation. Furthermore, we have modified the source code such that we can specify local, time‐dependent heat sources to force the GWs in the idealized model (see also SA15).

### 2.3 CPOL Radar Measurements

The C band polarimetric (CPOL) radar samples data with a volume scan out to a range of 150 km at a radar wavelength of 5.3 cm and peak power of 250 kW (Grimsdell et al., 2010; Keenan et al., 1998). The radar is located at Gunn Point (12.25°S,131.04°E), ∼20 km north of Darwin. One volume scan is completed every 10 min and its data are interpolated onto a Cartesian grid with 2 km horizontal and 1 km vertical resolution. Further information about the radar data is given in Grimsdell et al. (2010).

The depth and strength of heating are the two most important parameters determining GW propagation and amplitude (e.g., Alexander et al., 1995; Stephan et al., 2016). The parameters most representative of convective depth and magnitude from radar observations are echo top height and precipitation rate. The echo top height is estimated from radar measurements by calculating the altitude where the radar reflectivity decreases below 6 dBZ as was also done by Grimsdell et al. (2010). Precipitation rates are derived by using algorithms from Bringi et al. (2001) and Bringi et al. (2004) which take into account reflectivity, differential reflectivity, and specific differential phase. An extensive validation including uncertainty estimates of the radar precipitation rates can be found in Grimsdell et al. (2010).

### 2.4 AIRS Observations

Located on board the Aqua satellite (which is part of the A Train), AIRS orbits Earth with a period of 98.8 min crossing the equator at about 1:30 p.m. local solar time on ascending passes and 1:30 a.m. on descending passes. AIRS is a nadir‐looking instrument and scans the atmosphere perpendicularly to the satellite's ground track with a swath width of 1,780 km and a horizontal resolution at nadir of 13.5 km. AIRS data products include retrievals of atmospheric temperature; however, these standard retrievals include “cloud clearing,” which results in a loss of horizontal resolution and failure to resolve stratospheric GWs above deep convective clouds (Hoffmann & Alexander, 2010). Here we instead compare the full resolution AIRS brightness temperatures at 4.3 μm to simulated brightness temperatures, computed by filtering the simulated temperature profiles with the AIRS kernel function (Hoffmann & Alexander, 2010). We can then directly compare these model brightness temperatures with the AIRS brightness temperatures. The procedure is the same as in SA15.

The region examined in this study is near nadir where the horizontal resolution of the observations is about 14 km (Grimsdell et al., 2010). In the vertical, the AIRS kernel function width permits observation of waves with vertical wavelength longer than 12–13 km. Waves traveling upwind into a wind field of monotonic vertical shear are refracted to longer vertical wavelengths which are more visible to AIRS, while waves traveling downwind are less visible due to refraction to shorter vertical wavelengths. Since the horizontal background wind was mainly westward in the lower stratosphere above Darwin, we expect an east‐west asymmetry in the observed GW field caused by this asymmetry in visibility (Grimsdell et al., 2010; their Figure 1). Furthermore, the point in time of the AIRS observations relative to the time when GWs are convectively excited is important. As waves with different frequencies travel with different speeds, the timing of wave triggering relative to the overpass time of the satellite means that the comparison with the model is very sensitive to the timing, shape, and locations of GW sources.

## 3 Validation of Cloud‐Resolving Full‐Physics WRF Simulations

**Figure 3:**
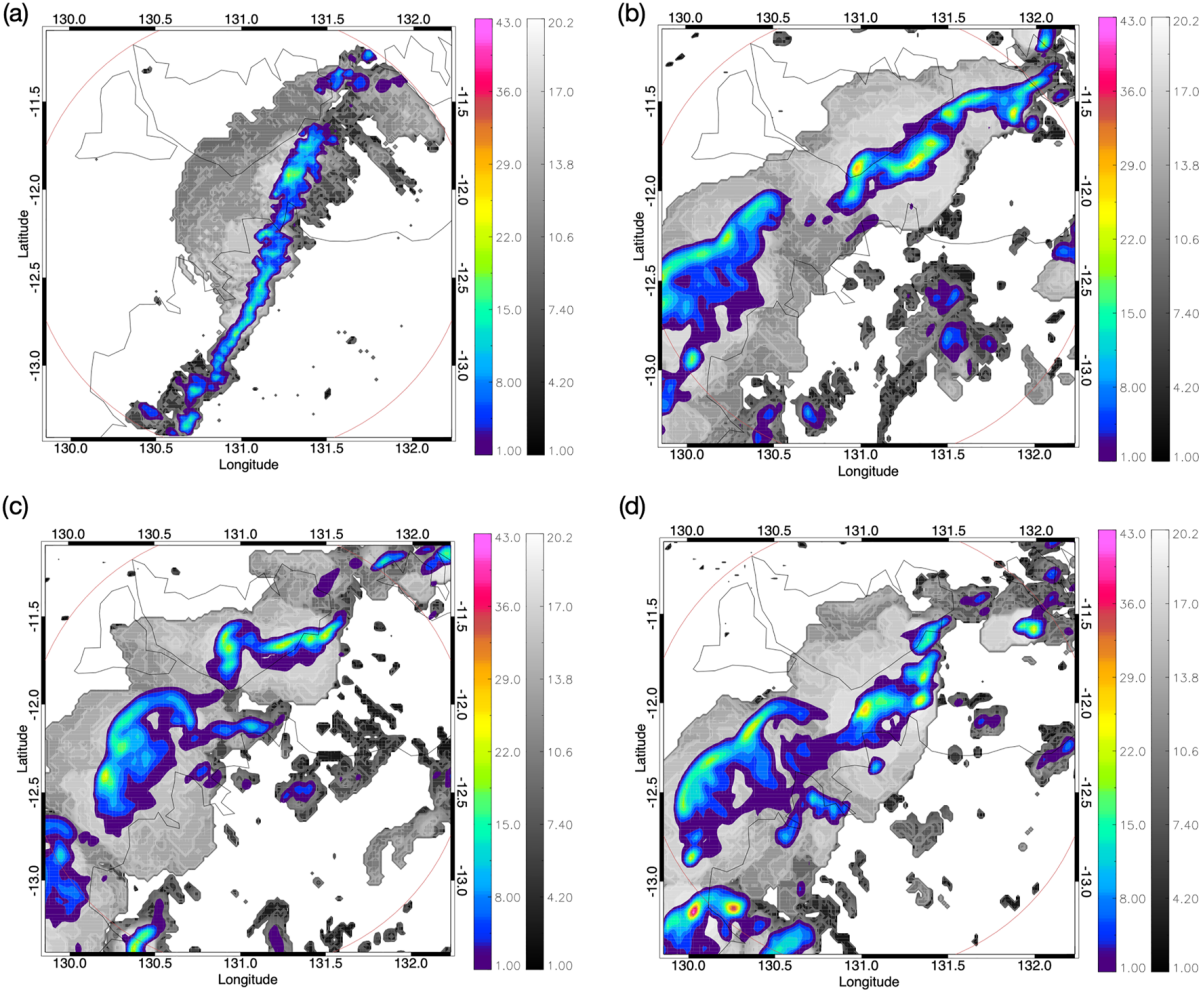
Composite of echo top height (in km, gray scale) and precipitation rate (in mm (10 min)^−1^, color) for 12 January 2003 at 12:40 UTC as observed by the C‐Pol radar (a) and simulated by WRF using a model top of 29.8 (b), 30, (c) and 32 km (d). The red circle shows the range of the radar, and the thin black lines show the coastline.

Figure 3 shows instantaneous echo top height (gray scale) and precipitation rates (color contours) at 12:40 UTC for radar observations (a) and for the inner domain of the three full‐physics WRF simulations (b–d). The horizontal grid spacing is 2 km in all panels. In this figure only precipitation rates larger than 1 mm (10 min)^−1^ are shown. Similar to the observations, the simulations show organization into a squall‐line storm which moves north‐west with time. Although we find the squall line sufficiently well represented in our simulations, it might still be partly underresolved at a grid spacing of 2 km. As is typical for cloud‐resolving simulations, the peak precipitation rates occur at different locations and times than in the observations, which is the reason we cannot compare gravity waves in these simulations directly to the single satellite overpass. Instead, we seek to realistically simulate a reasonably representative range of convective cell precipitation rates and echo top heights, which will then allow us to estimate latent heating directly from the radar. For this purpose, our focus is on comparing simulated and observed precipitation rates and echo top heights.

**Figure 4:**
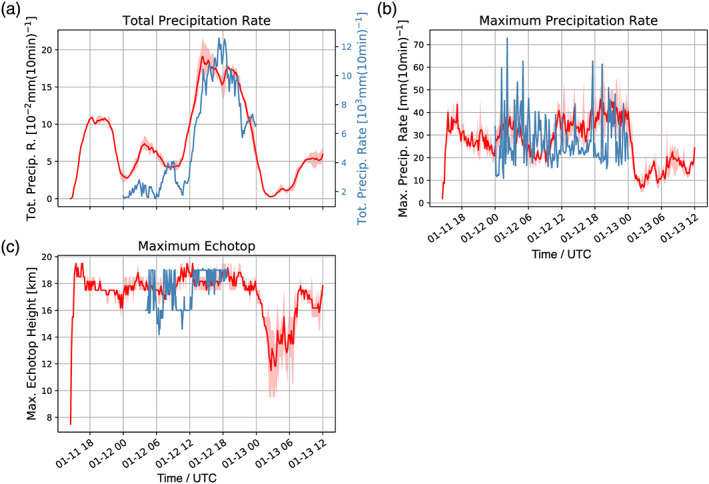
Time series of the total precipitation rate (a), the maximum precipitation rate (b), and maximum echo top heights (c) for convective pixels over the inner model domain and from C‐Pol radar observations. The red lines refer to simulated quantities where the shading highlights the spread of the different WRF simulations and the thick red line is the mean of the WRF ensemble. The blue line shows the radar‐observed quantities. Please note that the simulated total precipitation rate is shown per km^2^.

To further evaluate simulated and observed storm development, we next compare the temporal evolution of the precipitation rate and maximum echo top height for convective pixels which are defined as grid points with precipitation rates exceeding 1 mm (10 min)^−1^. Figure 4 illustrates the time series of the simulated total precipitation rate (Figure 4a), maximum precipitation rate (Figure 4b) and maximum echo top height (Figure 4c) for the radar and the inner domain of the WRF simulations with shading showing the range of the ensemble member values. The radar echo top heights have been prebinned with a vertical resolution of 1 km, where 19 km represents the uppermost value around which the bins are centered (so the true value can vary about ±0.5 km of the bin value). Both the observed and simulated time series show a distinct maximum in precipitation between about 14 and 21 UTC (Figure 4a). To account for the different areas simulated and observed we show the total precipitation rate per km^2^. The maximum precipitation rates show comparable magnitudes and evolution with time (Figure 4b), but with fewer extremes in the simulations. Overall, the temporal evolution of precipitation rates produced by the cloud‐resolving WRF simulations is similar to the 12 January 2003 observations. Also, the observed and simulated maximum echo top heights show comparable magnitudes and variability.

**Figure 5:**
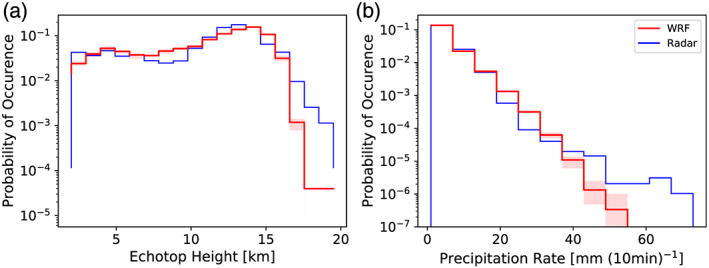
Probability density functions (in histogram style) of the echo top height (a) and precipitation rate (b). For the WRF PDFs only data at convective pixels within the radar measurement range are taken into account. Blue lines refer to C‐Pol radar observations and red line to WRF simulations, respectively. The shading shows the spread of the different WRF simulations.

To assess the capability of our cloud‐resolving WRF simulations to reproduce statistically similar ranges of precipitation and echo tops for convective rain, we compare distributions of these variables to the radar observations. Figure 5a shows probability distributions for the simulated and observed echo top heights, with values ranging from 2–19 km. For echo top heights ranging between 5 km and about 11 km the two agree well. However, the probability of occurence of cloud top heights lower than about 5 km is underestimated by the WRF simulations as are the occurrence of the deepest 19 km tops. The PDFs of echo top heights peak near 13 km in the observations and near 15 km in the simulations. Both observed and simulated echo top distributions suggest a double‐peaked structure, with one population near 5 km, and a second peak near 12 to 15 km.

The PDFs associated with the convective precipitation rates (Figure 5b) also suggest that the cloud‐resolving simulations reproduce the observations in a statistical sense fairly well. For precipitation rates smaller than 20 mm (10 min)^−1^ the PDFs of the observations and simulations match almost perfectly while the occurence of moderate precipitation rates is overestimated by the simulation (between 20 and 38 mm (10 min)^−1^). At precipitation rates larger than about 40 mm (10 min)^−1^ WRF simulations underestimate the probability of occurence compared to the observations and fail to simulate precipitation rates larger than 52 mm (10 min)^−1^ in the area of radar observations. For both the PDFs of echo top height and precipitation rates, the comparison shows good agreement, associated with shallow congestus and deep convective rain, respectively.

Overall the comparison between CPOL radar measurements and full‐physics WRF simulations indicates that our ensemble of simulations is capable of simulating the essential characteristics of the storm development and convective rain cell variability around Darwin, Australia on 12 January 2003. This gives us confidence that the ensemble of cloud‐resolving WRF simulations are sufficiently representing the variety of tropical convective GW sources for this case study.

## 4 Statistical Mean Properties of Latent Heat Profiles Deduced From Full‐Physics WRF Simulations

From the full‐physics WRF simulations, we next compute the statistical mean properties of latent heating profiles and evaluate their relation to precipitation rate and echo top height. For this analysis we use again the results of the innermost model domain (d03) and take into account convective rain pixels only (i.e., precipitation rates exceeding 1 mm (10 min)^−1^). Following SA15 we use the WRF *h*_*diabatic* latent heating field which is evaluated in the microphysics scheme and stored at the end of each time step. While SA15 used an idealized algorithm to form the latent heating profiles based on the single‐parameter precipitation rate, in this study we employ a lookup table approach which facilitates the extension of the method to include two parameters: precipitation and echo top height.

**Figure 6:**
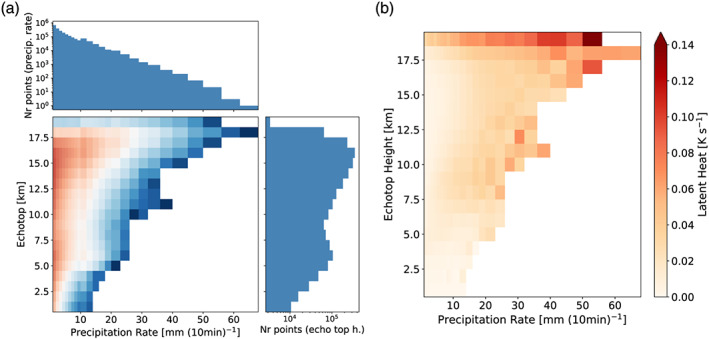
Histogram of echo top heights and precipitation rates as simulated by the three WRF runs for all convective pixels in the entire inner domain d03. In (a) the color coding refers to the number of occurrences which is given in the side panels. In (b) the color shading shows the maximum of the latent heat profile.

Figure 6a shows the histogram associated with echo top heights and precipitation rates for the complete ensemble of the full‐physics WRF simulations. The color shading in this figure is the number of points per bin (shown in the side panels). The bin size for the echo top height is 1 km to match the vertical resolution of the CPOL radar echo top heights. For the precipitation rate the bin size is 1 up to 10 mm (10 min)^−1^ and from there on increases by 1 mm (10 min)^−1^ per decade (i.e., 2 mm bins to 20 mm (10 min)^−1^, 3 mm bins to 30 mm (10 min)^−1^, etc.). We chose this approach to retain a fine a significant number of occurrences also at high precipitation rates.

The histogram relating precipitation rates and echo top heights shows that large precipitation rates are associated with high echo top heights (Figure 6a). It also illustrates that lower precipitation rates (≤5 mm (10 min)^−1^) are not associated with a specific echo top height range. The distribution of echo top heights at the higher precipitation rates is characterized by two distinct maxima at around 5 and 15 km. With a maximum of 62 mm (10 min)^−1^, the precipitation rates found in our study are larger than those associated with midlatitudinal squall lines (14 mm (10 min)^−1^) over the United States (Stephan & Alexander, 2015). In Figure 6b we relate the maximum in the profile of latent heating rate to echo top heights and precipitation rates. This analysis suggests that latent heating increases with both rising precipitation rates and echo top heights.

**Figure 7:**
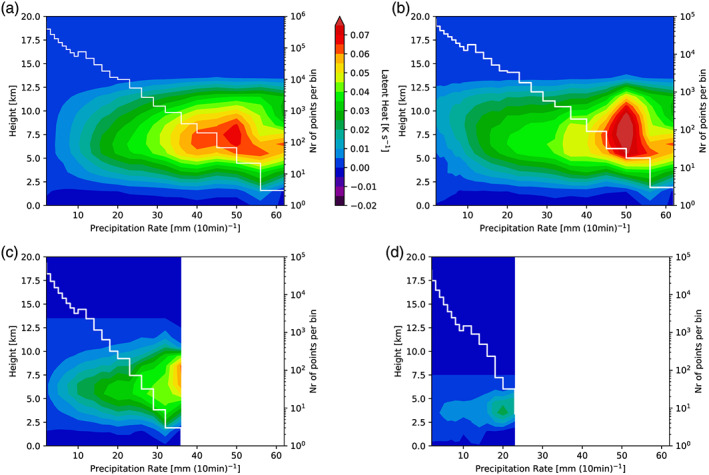
Simulated vertical latent heating profiles versus precipitation rate including all echo top heights (a). Panels (b) to (d) show the mean vertical heating profiles associated with echo top heights ranging from 13–19 (b), 7–13 (c), and 2–7 km (d), respectively. The vertical latent heating profiles are averages over the convective pixels of the inner domain d03 between 11 January 18 UTC to 13 January 12 UTC. White lines show the number of points for each precipitation rate bin (right‐hand axis).

Figure 7 presents the statistical mean profiles of latent heating where Figure 7a shows the profiles associated with precipitation rates only and Figures 7b–7d the profiles related to different ranges of echo top heights. Enhanced values of latent heating (i.e., >0.02 K s^−1^) are concentrated in an altitude range from 3 to 12.5 km. Again, our analysis indicates that the strongest latent heating (>0.06 K s^−1^) is associated with higher precipitation rates (>40 mm (10 min)^−1^) and deep convection (see Figure 7b). Relating the latent heating profiles additionally with the echo top height also enhances the maximum latent heating values for weaker deep convection. In particular, latent heating maxima increase by about 0.01 K s^−1^ for precipitation rates below 15 mm (10 min)^−1^ (compare Figure 7a to Figure 7b). Please note that the presented profiles in Figures 7b to 7d are mean profiles over the respective echo top heights mentioned in the caption. The true maximum of these profiles is 0.17 K s^−1^, higher than the maximum of 0.07 K s^−1^ for the profile related to precipitation rate only. We will discuss in section 5 how the differences in the profiles affect the excited GWs.

## 5 Gravity Wave Characteristics Derived From Idealized WRF Simulations

As already mentioned, we have modified the idealized WRF model such that the potential temperature field is forced by input heating. To determine this input heating, the statistical mean profiles of latent heating based on the full‐physics WRF simulations (Figures 6 and  7) are used as lookup tables. As the latent heating profiles are associated with precipitation rates and echo top heights, we can relate them to observed precipitation rates and echo top heights to derive three‐dimensional, time‐dependent input heating for the idealized WRF simulations. To derive these latent heating fields we first determine the closest observed precipitation rates and echo top heights to the respective bins and then interpolate within lookup table values.

In the following we analyze how the addition of the echo top height in the determination of the input heating profile affects the GW propagation characteristics and their amplitudes. For the remainder of this study the latent heating profiles associated with precipitation rates only (Figure 7a) are referred to as 1‐D lookup table while the profiles related to precipitation rates and echo top heights are called 2‐D lookup table (Figures 6 and  7b–7d).

### 5.1 Comparison to AIRS Observation

**Figure 8:**
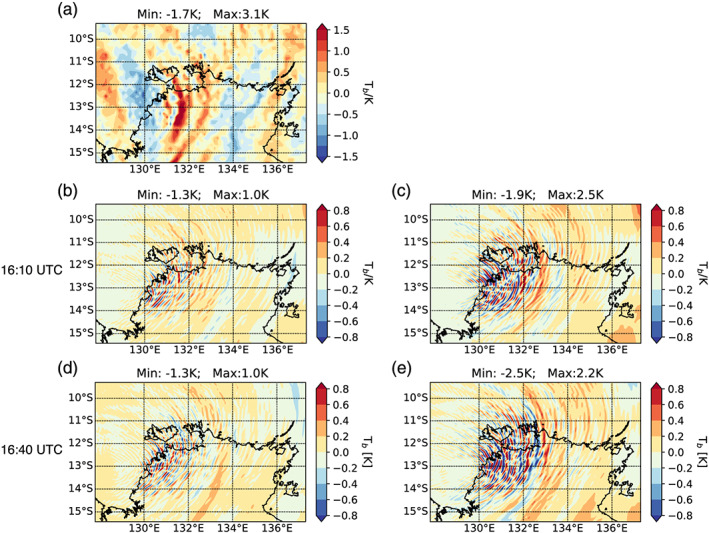
Measured and simulated brightness temperature perturbations. Panel (a) shows the brightness temperature perturbations derived from AIRS observations. Panels (b) and (d) illustrate the brightness temperature perturbations based on the dry idealized WRF runs initialized with 1‐D lookup table and panels (c) and (e) with the 2‐D lookup table, respectively. The simulated results are valid for 12 January 16:10 UTC (b and c) and 16:40 UTC (d and e), respectively, while the measurement was taken at 16:40 UTC.

Figure 8 shows observed (Figure 8a) and simulated (Figures 8b to 8e) brightness temperature perturbations. The AIRS kernel function filters all but the very long vertical wavelength GWs which are eastward due to the westward wind at this altitude (about 40 km). The brightness temperature perturbations derived from AIRS observations suggest a GW amplitude range of 4.8 K (between ‐1.71 and 3.13 K). The idealized WRF simulations generally reproduce the observed wave patterns, however, with clear differences. The amplitude range found with the 1‐D lookup table setup is 2.3 K (Figures 8b and 8d) about half the range seen in the observations. In contrast, the idealized WRF simulations based on the 2‐D lookup table reproduce the observed amplitude range, with 4.4 K at 16:10 UTC (Figure 8c) and 4.7 K at 16:40 UTC (Figure 8e), the time of the overpass. Thus, the comparison to AIRS observations clearly shows that including echo top heights in the generation of the lookup table leads to a consistently improved representation of the GW field in the simulations.

The observed temperature perturbations (Figure 8a) indicate a tendency for larger positive than negative perturbations which is not present in the simulated results. This might be related to the presence of a large‐scale wave originating outside of the domain. In our idealized simulations the forcing is based on the radar observations which means that only convective sources in the area of the radar observations can be captured. Thus, sources for large‐scale waves from outside the domain are not included in our simulations, which could lead to a different bias in the simulated brightness temperature perturbations.

### 5.2 Momentum Flux, Phase Speed, and Propagation Direction

**Figure 9:**
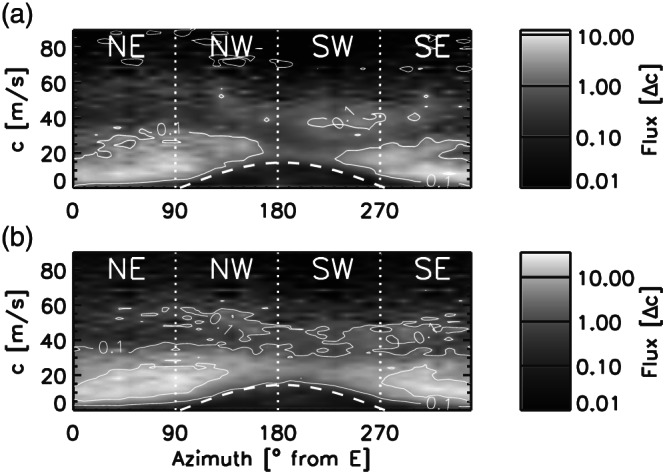
Wave momentum fluxes versus propagation direction and ground‐based phase speed derived from idealized simulations initialized with the 1‐D lookup table (a) and 2‐D lookup table (b), respectively. The thin white line denotes the contour of the wave momentum flux at 0.1 and 1.0 · 10^−6^ Pa (m s^−1^)^−1^. Momentum fluxes are calculated over a period of 4 hr starting at 12 January 13 UTC, and the figures show the mean momentum flux over an altitude range from 19 to 25 km. The dashed white line overplotted on the momentum fluxes shows the vector stratospheric wind that filters an arc‐shaped region of phase speed. Note that flux units are given per unit phase speed in 10^−6^ Pa (m s^−1^)^−1^ and the range of the color scale in the two panels differs.

Figure 9 shows total horizontal momentum flux (MF) spectrum as a function of phase speed and propagation direction at levels in the lower stratosphere as already done in Alexander et al. (2006). Note that the gray‐scale shadings refer to different scales in panels (a) and (b). These MFs were computed over a time period of 4 hr starting at 13 UTC. Overall, the GWs simulated by the 2‐D lookup table setup (Figure 9b) transport more horizontal MF than the waves generated by the setup initialized with the 1‐D lookup table (Figure 9a). The maximum magnitude of the total MFs contained in the GWs excited by the 2‐D lookup table setup is 36.1 mPa, while the maximum MF simulated by the 1‐D lookup table setup is 12.3 mPa.

**Figure 10:**
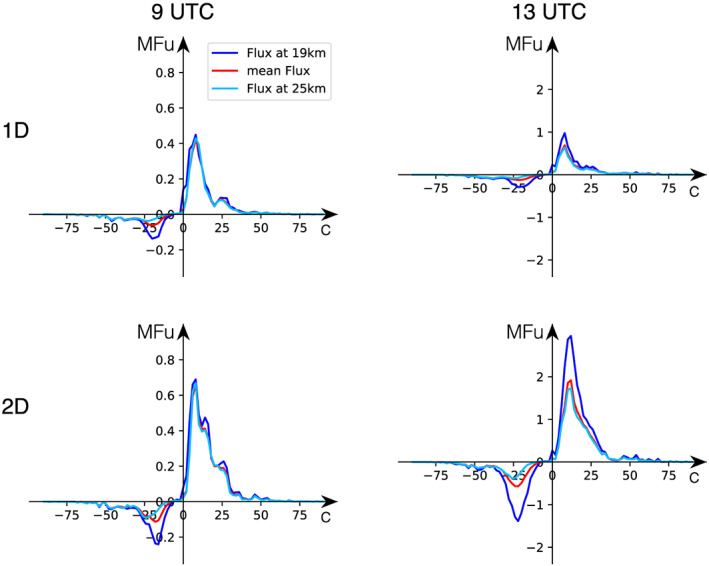
Zonal momentum flux versus phase speed based on idealized WRF simulations initialized with the 1‐D lookup table (upper row) and 2‐D lookup table (lower row). Momentum fluxes are calculated over a period of 4 hr starting at 12 January 9–13 UTC (left column) and 13–17 UTC (right column). The phase speed shown is the projected phase speed onto the east west direction. Flux units are given per unit phase speed in 10^−4^ Pa (m s^−1^)^−1^. The red curves show average flux 19–25 km altitude, while the dark and light blue curves show 19 and 25 km, respectively.

As the QBO is affected by the deposition of zonal momentum of GWs and for our case the main wind direction in the lower stratosphere is westward, we next examine the distribution of the zonal MF with phase speed at different altitudes. For this analysis the zonal MF is integrated over propagation directions with the phase speed projected onto the zonal direction. In Figure 10, the distribution of zonal MF shows that eastward propagating GWs contain up to 2.5 times more horizontal MF than the westward propagating GWs in both idealized simulation setups. The zonal wind was directed westward in the lower stratosphere above Darwin (Figure 2) which could explain the larger MF transported by eastward propagating GWs as the westward propagating GWs are filtered by the background wind. The phase speed associated with the maximum MFs is about 10 m s^−1^ for eastward propagating GWs and 20 to 25 m s^−1^ for westward propagating GWs.

Two different time periods are shown in Figure 10: 9–13 UTC representing the developing phase of the squall line, and 13–17 UTC representing the mature phase. The zonal MFs simulated using the 2‐D lookup table are larger than those based on the 1‐D lookup table setup especially at 13–17 UTC. During the mature phase the fluxes from the 1‐D lookup table setup are only up to one third of the fluxes derived from the 2‐D lookup table setup. Furthermore, the temporal evolution of MF magnitudes is different between the two idealized simulation setups. While the magnitudes of zonal MFs double for the 1‐D lookup table setup, these fluxes increase on average by a factor of about 3 between 9–13 and 13–17 UTC in the simulations initialized with the 2‐D lookup table setup.

### 5.3 Zonal Forcing on Background Flow

**Figure 11:**
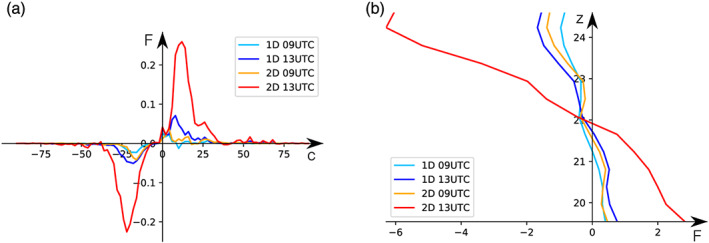
Panel (a) shows zonal forcing between 19 and 25 km altitude as a function of phase speed. Again, the phase speed is the projected phase speed onto the east‐west direction. Forcing units are given per unit phase speed in m s^−1^ day^−1^ (m s^−1^)^−1^. Panel (b) shows the net zonal force per unit mass estimated from the convergence of the zonal momentum flux exerted on the ambient flow by the simulated GW activity. Here, the force unit is given in m s^−1^ day^−1^. For both panels blue colors refer to results from idealized WRF simulations using the 1‐D lookup table, those using the 2‐D table are red colors. Light blue and orange show results from 9–13 UTC and dark blue and red show 13–17 UTC.

Figure 11a shows the zonal forcing between 19 and 25 km as a function of phase speed. Generally, linearly propagating GWs conserve vertical flux of horizontal momentum with altitude which means that zonal MF divergence indicates the deposition of momentum to the ambient flow. In concurrence with Figure 10 we find the highest zonal forcing for the 2‐D lookup table setup for the mature phase of the squall line (denoted as 2‐D 13 UTC). For both the 1‐D and 2‐D lookup tables the peak forcing is concentrated at phase speeds between 0 and 30 m s^−1^ in both the eastward and westward directions for the mature phase.

The net force acting on the background flow due to the deposition of zonal MF is presented in Figure 11b. Following Alexander and Holton (1997) this force has been corrected by the GW energy leaving the simulation domain. However, as this effect is found to be orders of magnitude smaller than the GW force, we will not discuss it in more detail (not shown). With a maximum eastward force in the plane of GW propagation of up to about 2.5 m s^−1^ day^−1^ and a westward force of up to about 6 m s^−1^ day^−1^ the impact of the net force on the ambient flow is significant. However, we have modeled only one region containing a strong convective storm, so in order to compare these numbers to zonal‐mean, time‐mean forces driving the QBO, one has to keep in mind that these forces are based on MFs calculated over a time period of only 4 hr and over a domain width of only 700 km. Therefore, the values given in this analysis would be expected to be larger than GW forcing on the zonal‐mean ambient flow. Furthermore, the forcing profiles in Figure 11b suggest for all realizations that the impact of the forcing on the ambient flow changes with altitude. Below 22 km the deposition of the zonal momentum flux leads to an eastward force, while above 22 km it is westward. This is likely associated with the shear, which is eastward at ∼16–21 km switching to westward above (Figure 2a).

The excited net force on the ambient flow is largest at 13–17 UTC for the idealized WRF setup initialized with the 2‐D lookup table (see Figure 11b). Also, the temporal evolution is more pronounced for the 2‐D lookup table setup than for the 1‐D one. While the simulated forces associated with the 1‐D lookup table setup exhibit no significant temporal evolution, the net forces related to the 2‐D lookup table setup increase by up to a factor of about 5 at an altitude of about 24 km.

## 6 Discussion

Our analysis shows similar distributions and magnitudes of zonal MF from the 1‐D and 2‐D simulation between 9 and 13 UTC (Figure 10). However, the magnitudes increase more rapidly with time in the simulation initialized with the 2‐D lookup table where the zonal MF becomes up to 3 times larger than the one associated with the 1‐D lookup table. It appears that initially the GWs simulated by the two different setups are comparable; however, the differences in the GW characteristics increase over time.

**Figure 12:**
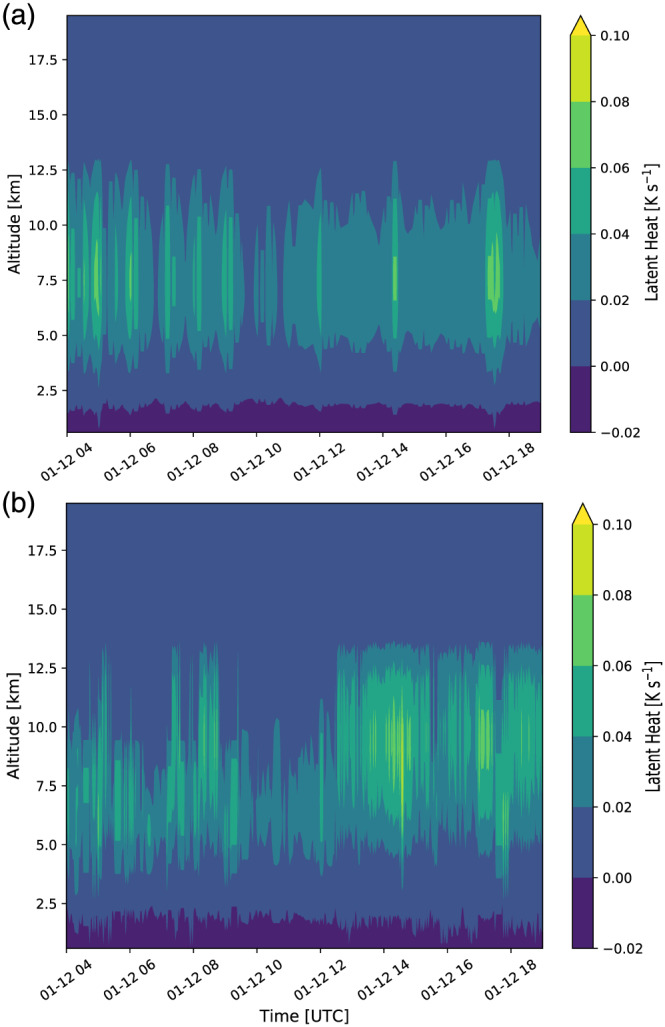
Time series of vertical profiles at the latitude/longitude location of maximum latent heat for the idealized simulations initialized with the 1‐D lookup table (a) and 2‐D lookup table (b).

We analyze the temporal evolution of the vertical profiles of latent heat based on Figure 12 to study a possible cause for the different temporal evolution of GW characteristics in the two different idealized WRF simulations. In this context we want to highlight three major differences: (1) the maxima of latent heating associated with the 2‐D lookup table are higher than those related to the 1‐D lookup table (particularly after 12:30 UTC on 12 January); (2) the vertical extent of the latent heating is larger in the 2‐D lookup table setup by about 2 km between 7 and 8 UTC as well as after 12:30 UTC; (3) the temporal evolution of input heat based on the 1‐D lookup table is much more uniform than for the 2‐D lookup table. Generally, higher input latent heat values lead to larger‐amplitude GWs which consequently transport higher zonal MFs. This could explain the higher zonal MFs especially starting at 13 UTC for the simulations initialized with the 2‐D lookup table. Our analysis clearly shows that including the echo top height in the development of the lookup table leads to significant changes of GW characteristics which can in turn also alter their impact on the background atmospheric flow in simulations.

In our study the zonal MFs peak at phase speeds between 0 and about 30 m s^−1^ in the eastward and westward direction. There are limited observations to which we can compare these results. MFs peak at lower phase speeds, between 0 and about 15 m s^−1^, in observations shown in Pfister et al. (1993) and Jewtoukoff et al. (2013). Jewtoukoff et al. (2013) stated that a numerical simulation of a cyclonic storm case tends to overestimate the higher phase speeds compared to observations. Recently, Kang et al. (2017) applied a GW parametrization to study climatological GWs spanning 32 years and reported zonal MFs at 15° latitude exhibit a pronounced maxima between about 5–20 m s^−1^ in the eastward direction and 5–10 m s^−1^ in the westward direction, respectively. While our eastward zonal MF distribution compares well to this estimate, the distribution of our westward MF is larger at higher phase speeds. However, these differences might also be due to the nature of the GWs generated by this one regional case study.

Several numerical studies showed that GW absorption provides a driving force for the QBO (e.g., Alexander & Holton, 1997; Kawatani et al., 2010; Lindzen & Holton, 1968; Piani et al., 2000). Although the GWs taken into account in our study are launched near the edge of the equatorial region, these GWs nevertheless may be indicative of other deep convective sources closer to the equator (Holton et al., 1995). Already (Piani et al., 2000) studied the impact of convectively generated GWs on the mean atmospheric circulation near Darwin, Australia. In their study they used a 3‐D mesoscale model and found maximum forcing of 1 m s^−1^day^−1^. With a maximum of about 6 m s^−1^day^−1^ the forcing found in our study is significantly larger than in Piani et al. (2000). However, compared to Alexander and Holton (1997) who used a 2‐D modeling approach, the forcing is a similar order of magnitude. The difference to the results of (Piani et al., 2000) may be related either to the strength of the excited convective GWs or the horizontal wind profiles. While Alexander and Holton (1997) used a horizontal wind profile with a shear layer of similar magnitude at the same altitude region as analyzed in our study, the shear layer is located at higher altitudes and of smaller magnitude in Piani et al. (2000).

Observational and numerical modeling studies suggest that the zonal mean forcing necessary to drive the QBO is about 0.3 – 0.4 m s^−1^day^−1^ (e.g., Anstey et al., 2016; Ern et al., 2014; Garcia & Richter, 2019; Holt et al., 2016; Kawatani et al., 2010; Kim & Chun, 2015; Richter et al., 2014). The approximate areal coverage of the storm analyzed in our study is about 0.6% of the total area equatorward of ± 20° latitude. The maximum forcing related to the 2‐D lookup table setup is on the order of 6 m s^−1^day^−1^. Thus, if 15 such tropical storms were present in the entire equatorial region they could account for 15% to 20% of the forcing required for downward propagation of the shear zones related to the QBO. In former studies, a contribution of convective GW momentum flux divergence up to about 50% was found depending on the phase of the QBO (Alexander & Vincent, 2000; Piani et al., 2000). Results with global models set up with high‐resolution vertical grids suggest that GWs with wavelengths smaller than 1,000 km contribute substantially to the small‐scale momentum flux divergence with magnitudes up to 50% in the eastward phase and 55–70% for the westward phase of the QBO (Holt et al., 2016). Furthermore, long‐term high‐resolution satellite observations suggest that for both QBO phases the strongest 10% GW events account for more than 35% of the total observed momentum fluxes at 20 km altitude (Ern et al., 2014). Our results indicate lower forcing compared to recent studies. However, we analyzed GWs related to only one strong convective storm where the derived forcing represents an extrapolation of a 4‐hourly value to a complete day which can lead to an underestimation of the GW induced forcing in our case.

## 7 Summary and Conclusions

In the presented study we extended the approach of SA15 to a tropical storm system. SA15 had developed a heating algorithm for the realistic simulation of gravity waves (GWs) specifically excited by midlatitude mature continental storm systems during summer time. Due to the different environmental conditions in our study the lookup tables we present are significantly different to the table in SA15 (see their Figure  3). Both, the precipitation and latent heating rates found in our study are larger by a factor of about 5 and 10, respectively. Due to the deeper convection in the tropics the altitude range covered by enhanced latent heating rates (i.e., >0.02 K s^−1^) increases by about 5 km. Despite these marked differences between the midlatitudinal and tropical lookup tables overall we show that the method is applicable also to tropical conditions.

Furthermore, we developed the lookup table not only as a function of precipitation rate but additionally of cloud top height. In our study we showed that this extension affects the lookup tables (Figures 6, 7) as the maximum latent heating rates are associated with echo top heights larger than 13 km (see Figure 6). These differences caused a different representation of the convective GW field in the idealized WRF simulations where the 2‐D lookup table setup resulted in an increase in GW amplitudes by about a factor of 2 (see Figures  8 and 10). Also, the temporal evolution of the GW field seems to be affected by expanding the lookup table to the echo top height. Overall, we conclude that the extension of the lookup table to the echo top height proved to be valuable by leading to a more realistic representation of the convective GW field especially regarding their amplitude range. That way our method could also be used to improve the representation of convective gravity waves in climate and weather models.

In our study we showed that the method of SA15 can be extended to a tropical storm system above Darwin, Australia. However, further study is required to analyze whether this method is also applicable to other more equatorial locations within the tropics and intertropical convergence zone (ITCZ). Also, an open question remains whether there is a difference between the lookup tables related to convection over land mass or oceans. Furthermore, we plan to include the stage of convection (developing or mature convection) in the development of the lookup table as a third variable. For more general applications there is also the question whether the lookup table has a seasonal dependency.

We have already mentioned that only limited observations of convectively generated tropical GWs exist to date to which modeling results can be compared. In this context long‐duration flights of superpressure balloons are conducted in the Strateole 2 campaign. Equipped with high‐resolution instruments, these balloon flights will lead to dramatic new insights into GW generation by convection and wave effects on the mean flow. That way the data will permit detailed validation of future similar models and further new developments.

## Data Availability

The gravity wave data sets used in this study have been created using AIRS/Aqua L1B Infrared (IR) geolocated and calibrated radiances V005 distributed by the NASA Goddard Earth Sciences Data and Information Services Center (GES DISC). They can be accessed at https://datapub.fz-juelich.de/slcs/airs/gravity_waves or by contacting Lars Hoffmann, Juelich.
